# Towards automated on-line adaptation of 2-Step IMRT plans: QUASIMODO phantom and prostate cancer cases

**DOI:** 10.1186/1748-717X-8-263

**Published:** 2013-11-08

**Authors:** Kostyantyn Holubyev, Klaus Bratengeier, Mark Gainey, Bülent Polat, Michael Flentje

**Affiliations:** 1 Klinik und Poliklinik für Strahlentherapie, Universitätsklinikum Würzburg, Würzburg, Germany; 2 Radiologische Klinik, Klinik für Strahlenheilkunde, Universitätsklinikum Freiburg, Freiburg, Germany

**Keywords:** Prostate carcinoma, IMRT, IGRT, Adaptation

## Abstract

**Background:**

The standard clinical protocol of image-guided IMRT for prostate carcinoma introduces isocenter relocation to restore the conformity of the multi-leaf collimator (MLC) segments to the target as seen in the cone-beam CT on the day of treatment. The large interfractional deformations of the clinical target volume (CTV) still require introduction of safety margins which leads to undesirably high rectum toxicity. Here we present further results from the 2-Step IMRT method which generates adaptable prostate IMRT plans using Beam Eye View (BEV) and 3D information.

**Methods:**

Intermediate/high-risk prostate carcinoma cases are treated using Simultaneous Integrated Boost at the Universitätsklinkum Würzburg (UKW). Based on the planning CT a CTV is defined as the prostate and the base of seminal vesicles. The CTV is expanded by 10 mm resulting in the PTV; the posterior margin is limited to 7 mm. The Boost is obtained by expanding the CTV by 5 mm, overlap with rectum is not allowed. Prescription doses to PTV and Boost are 60.1 and 74 Gy respectively given in 33 fractions.

We analyse the geometry of the structures of interest (SOIs): PTV, Boost, and rectum, and generate 2-Step IMRT plans to deliver three fluence steps: conformal to the target SOIs (S0), sparing the rectum (S1), and narrow segments compensating the underdosage in the target SOIs due to the rectum sparing (S2). The width of S2 segments is calculated for every MLC leaf pair based on the target and rectum geometry in the corresponding CT layer to have best target coverage. The resulting segments are then fed into the DMPO optimizer of the Pinnacle treatment planning system for weight optimization and fine-tuning of the form, prior to final dose calculation using the collapsed cone algorithm.

We adapt 2-Step IMRT plans to changed geometry whilst simultaneously preserving the number of initially planned Monitor Units (MU). The adaptation adds three further steps to the previous isocenter relocation: 1) 2-Step generation for the geometry of the day using the relocated isocenter, MU transfer from the planning geometry; 2) Adaptation of the widths of S2 segments to the geometry of the day; 3) Imitation of DMPO fine-tuning for the geometry of the day.

**Results and conclusion:**

We have performed automated 2-Step IMRT adaptation for ten prostate adaptation cases. The adapted plans show statistically significant improvement of the target coverage and of the rectum sparing compared to those plans in which only the isocenter is relocated. The 2-Step IMRT method may become a core of the automated adaptive radiation therapy system at our department.

## Background

For prostate cases, the clinical target volume (CTV) typically features large and random interfractional motions and deformations due to filling in the neighboring rectum and bladder [1-3]. Safety margins are introduced in the IMRT protocols to account for these motions. The margins, however, lead to undesirably high rectum toxicity [4]. The direct compensation of CTV motion using IMRT in combination with portal imaging devices (image-guided IMRT) is the way to reduce safety margins and to allow further dose escalation to the target and better sparing of critical structures. Two main approaches to image-guided IMRT are being pursued. As the relative movements in the constellation prostate-rectum-bladder has been shown to be patient-specific [2], one can accumulate patient statistics during the initial treatment fractions and use this information to derive a patient-specific CTV associated with reduced safety margins [5]. An alternative is the aperture-based image-guided adaptation. Some authors investigate the way to deform dose distributions [6] or intensity maps [7,8] according to the beam eye view (BEV) projections of structures of interest (SOIs) on the day of treatment; the linear programming model is then solved to translate dose distributions/intensity maps into adapted multileaf collimator apertures (MLC segments). Others propose to use the geometry of the day to directly restore conformity of MLC segments to the target [9-12]. Although the results of the latter studies are encouraging, simply restoring the segment conformity to the target does not account for the fundamental structure of IMRT plans. The analysis of the roles of IMRT segments [13] led the second author (KB) to propose a method of generating adaptable IMRT plans, which was termed “2-Step segmentation”. The associated set of adaptation rules was termed “2-Step adaptation”. The first result of manual 2-Step adaptation for the prostate patients followed [14].

In this paper we present the first result of the automated 2-Step adaptation of the IMRT plans. We start with a brief recapitulation of the principles of 2-Step adaptation. The adaptation to the changed geometry of the QUASIMODO phantom [15] is considered for illustrative purposes. Then the adaptation results for the prostate patients in the experimental environment closely resembling that of [14] are presented, discussed, and conclusions are drawn.

## Methods

The treatment planning system (TPS) Pinnacle^3^™ version 9.2 (Philips, Fitchburg, WI) was used in the present study for all dose calculations and plan optimisations. The Elekta Synergy S™ linac (Elekta AB, Stockholm, Sweden) with the multileaf collimator (MLC) with 4 mm leaves (BeamModulator™) has been commissioned in the TPS.

### 2-Step adaptation illustrated using QUASIMODO und prostate case

As was discussed in [16], the adaptation methods which preserve Monitor Units (MU) of MLC segments constant have a number of advantages. These methods assume that the interfractional geometry change is “small enough”, so that the segments may retain their original MUs and only their form needs to be adapted. The definition of “small” is not available beforehand, and has to come from practical experience. The introduced dose inhomogeneities can be compensated by additional shifts of MLC leaves. In principle, for any given number of MUs we can improve the homogeneity of the plan by shifting the MLC leaves, unless the interfractional geometry change is so large that under the condition of MU preservation there is no chance to arrive at a clinically acceptable plan. For quality assurance purposes, due to fewer degrees of freedom the plans adapted under MU preservation require fewer dosimetric pre-treatment checks than the plans resulting from MU-modifying adaptation. In conclusion, MU-preserving adaptation is an appropriate starting point in the development of any general adaptation technique.

The fundamental principles of generation of 2-Step IMRT plans are reviewed in the Appendix A; the references to the relevant literature are provided therein. It has been shown in a number of publications [13,14] that 2-Step IMRT plans intrinsically allow MU-preserving ad-hoc geometric adaptation due to clear functional classification of segments. The adaptation rules have been described in detail in earlier publications [13,14,17]. We illustrate them here briefly using the QUASIMODO phantom and the prostate cancer case.

**Figure 1:**
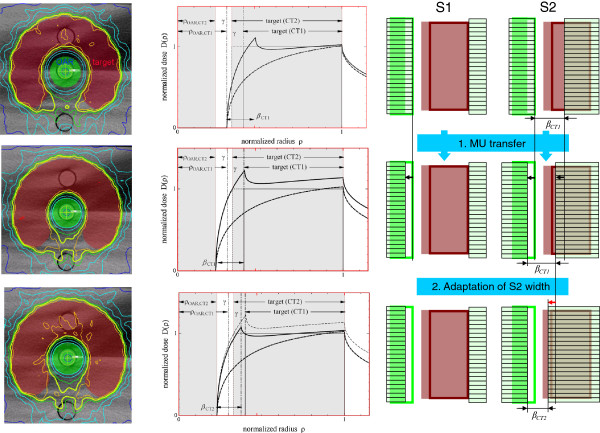
**Adaptation of S2 width.** Top – the CT1 plan applied “as is” underdoses inner areas of the CT2 target; middle – new 2-Step segments overdose the CT2 target under MU preservation; bottom – adaptation of S2 width restores required target coverage under MU preservation. The light-green line shows D_95%_ isodose.

The QUASIMODO phantom was introduced to compare coplanar IMRT techniques across institutions [15]. It represents a prostate cancer case with virtually no vertical change, but quite challenging in axial plane due to almost fully enclosed target. QUASIMODO geometry is also advantageous since it is close to the ideal geometry considered in [18] and [13]. Let’s consider two QUASIMODO geometries, planning geometry (CT1), and geometry of the day (CT2), where the target extends inwards from CT1 to CT2, and the gap γ between the target and the OAR remains constant, see Figure 1. The difference of the normalized OAR radii Δ*ρ* = |*ρ*_OAR, CT1_ - *ρ*_OAR, CT2_| is chosen to be of the order of 5%, which results in a 2% difference between planned MUs for the two geometries. According to our experience, such MU difference is typical for the interfractional geometry changes observed for prostate patients.

**Figure 2:**
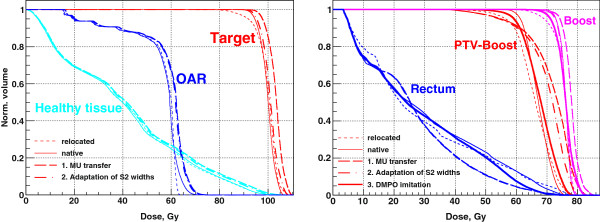
**2-Step adaptation for the QUASIMODO case (left) and for the real clinical case of prostate cancer treatment (right).** Thin dashed line – native CT1 plan applied to the CT2 geometry (relocated plan for the clinical case), thin solid line – native plan for the CT2 geometry (“golden standard”). The numbers denote adaptation steps. For the QUASIMODO case two adaptation steps are shown: 1. Generation of the 2-Step segments for the CT2 geometry with MUs transferred from the CT1 plan; 2. Adaptation of S2 widths according to (1). For the real clinical case additional step is performed: 3. Imitation of DMPO shifts for CT2 segments.

For the adaptation from CT1 to CT2, we want to achieve the target coverage not significantly worse than the coverage from the native CT2 plan. We build on the standard clinical protocol and relocate the isocenter of the native CT1 plan to have maximum target coverage in CT2. This is trivial in the case of the QUASIMODO phantom, where the necessary shift from CT1 to CT2 is zero by definition, but becomes important for real clinical cases. For the QUASIMODO phantom, the native CT1 plan relocated to CT2 underdoses inner areas of the target (Figure 1, top) which is reflected in the corresponding DVH, see Figure 2, left. Apparently, the segments need to follow the inward movement of the OAR edge. After new 2-Step segments are generated for CT2 and the MUs are transferred from CT1 (Figure 1, middle), the dose gradient moves back into the gap between the OAR and the target, but the target is overdosed (Figure 2, left, adaptation step 1). It has been shown [13] that one can restore the required target coverage under the condition of MU preservation if one additionally adapts the width of the narrow S2 segment to the changed geometry according to

(1) $$\beta_{\mathrm{CT}2}=\beta_{\mathrm{CT}1}\cdot \frac{\beta_{\gamma_{\mathrm{CT}2}}^{*,\text{adapt}}\left(\rho_{\text{OAR},\text{CT}2}\right)}{\beta_{\gamma_{\text{CT}1}}^{*,\mathrm{adapt}}\left(\rho_{\text{OAR},\text{CT}1}\right)}$$

where $\beta^{*,\mathrm{adapt}}:=\omega_{\mathrm{opt}}^{\mathrm{adapt}}\cdot \beta_{\mathrm{opt}}^{\mathrm{adapt}}$, $\left[\omega_{\mathrm{opt}}^{\mathrm{adapt}},\beta_{\mathrm{opt}}^{\mathrm{adapt}}\right]$ is the pair of normalized weight and width of the S2 segment which optimally restores required target coverage, minimizing objective function (A.1), see Appendix A, under the condition of MU preservation, *ω*_CT2_ = *ω*_CT1_. The quantity $\beta_{\gamma }^{*,\mathrm{adapt}}\left(\rho_{\mathrm{OAR}}\right)$ was measured on alternating geometries of the QUASIMODO phantom for different normalized gaps *γ* and normalized OAR radii *ρ*_OAR_, and parameterized using a polynomial of the fourth order [14,16]. This parameterization can be used to adapt S2 width of the day, *β*_CT1_, to the new geometry in every CT layer, assuming MU preservation, *ω*_CT2_ = *ω*_CT1_.

The result of adaptation of the S2 width is shown in Figure 1, bottom. According to [14], decreased OAR radius requires narrower S2 segments to remove the overdosage and restore required target coverage. The corresponding improved DVH is shown in Figure 2, left, adaptation step 2.

Having considered the idealized QUASIMODO phantom, we now move on to consider a clinical prostate cancer case. We use the relocated isocenter to generate 2-Step segments for CT2. In this way we ensure that approximately the same part of the BEV is used to irradiate the target in CT1 and in CT2, and the adverse effect of non-flatness inherent in the clinical beam profile is minimized.

In Figure 2, right, the DVHs for the real prostate case are shown. Boost and PTV - Boost are the target SOIs, their exact definition is explained in the next section. For now, it is important to have them both adequately covered, ideally approaching the “golden standard” of the native CT2 plan. Again, we consider the geometric difference between two CTs to be “small”, resulting in 1) a small difference between planned MUs, on the order of one per cent; 2) a similar 2-Step segment sets for the two geometries, with approximately the same number of 2-Step segments of each type. In Figure 2, right, adaptation step 1 corresponds to the 2-Step plan generated for the CT2 geometry with MUs transferred from the corresponding segments of the CT1 geometry. Adaptation step 2 shows the result of the adaptation of the widths of S2 segments. For each MLC leaf pair we find a corresponding CT slice, measure target and OAR radii and the OAR-target gap as projected in BEV, and calculate normalized values *ρ*_OAR_ and *γ* to be used in (1). As we see, the result for the Boost is quite encouraging, but not for the PTV - Boost.

**Figure 3:**
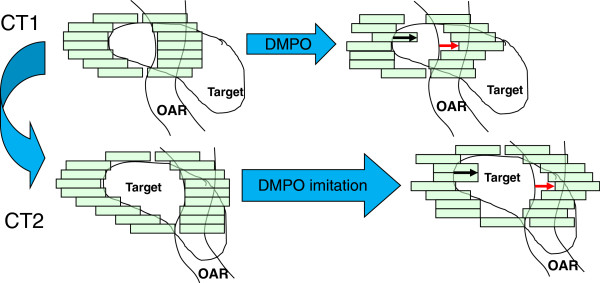
DMPO imitation: the DMPO fine-tuning for CT1 (blue arrow – sparing, red arrow – exposure) is imitated for CT2, taking changed vertical extension of the target into account.

Hitherto the deformations of the target SOIs (expansion/contraction, rotations) have been taken into account via the forward planning for CT2. However, the MUs found for CT1 segments cannot be separated from the features of the geometric form introduced by DMPO into CT1 segments. These features are imitated for CT2 segments, see Figure 3. We consider CT1 and store the shifts of the MLC leaves introduced to pre-DMPO 2-Step segments (Figure 3, top left) at the DMPO optimization stage (Figure 3, top right ). Then we consider CT2, find a corresponding pre-DMPO 2-Step segment (Figure 3, bottom left), and shift the MLC leaves the same distance and direction (Figure 3, bottom right). The changed vertical extension of the target is taken into account to have approximately the same portion of the BEV projection of the target exposed/spared in CT2 and CT1. The resulting adapted plan (Figure 2, right, adaptation step 3) provides target coverage improved over the relocated plan for both target SOIs.

To summarize, 2-Step adaptation adds three further steps to the standard clinical protocol of isocenter relocation:

1) 2-Step generation for the geometry of the day using the relocated isocenter, MUs are transferred from the planning geometry;

2) Adaptation of S2 widths to the geometry of the day for every MLC leaf pair according to (1). Normalized OAR radius *ρ*_OAR_ and normalized OAR-target gap γ are measured in the corresponding CT layer;

3) Imitation of DMPO fine-tuning for the geometry of the day.

### 2-Step adaptation: intermediate/high risk prostate carcinoma

Intermediate/high-risk prostate carcinoma cases are treated at UKW using Simultaneous Integrated Boost (SIB) [19]. Based on planning CT, a CTV is defined as the prostate and the base of seminal vesicles. The CTV is expanded by 10 mm resulting in the PTV, the posterior margin is limited to 7 mm. The Boost is obtained by expanding the CTV by 5 mm, overlap with rectum is not allowed. Prescription doses to PTV and Boost are 60.1 and 74 Gy respectively, given in 33 fractions [20].

**Figure 4:**
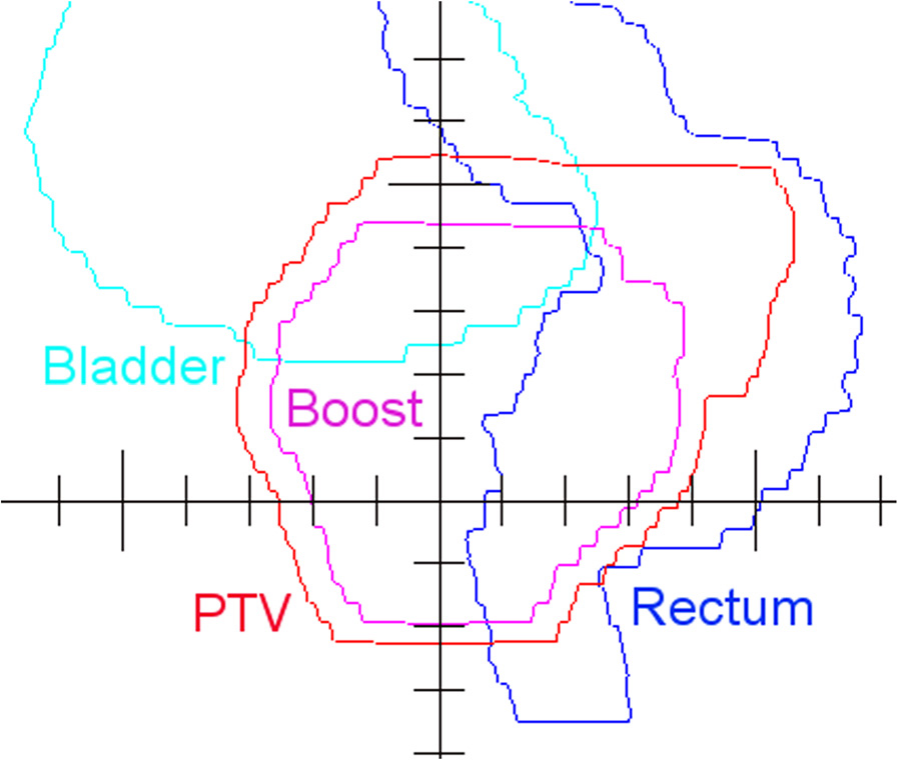
The BEV view of the SOIs.

**Figure 5:**
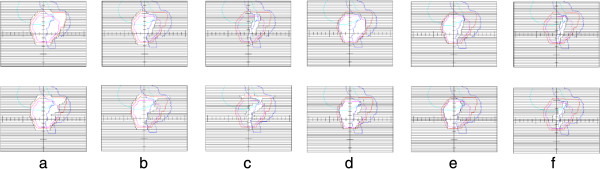
The pre-DMPO (top) and DMPO fine-tuned segments (bottom) oriented at the PTV (a-c) and at the Boost (d-f).

The 2-Step segments were generated using the in-house C++ software and fine-tuned in DMPO™ module of Pinnacle™ 9.2 in 25 optimization steps using a composite objective value (COV) as a function to be minimized. The COV is the sum of all weighted objective values. Objective values were defined as volume-normalized quadratic penalties referred to points in the dose volume histograms (DVHs). Typically four objectives were used for the PTV: two objectives described the requirements near the minimal dose and two near the maximal dose. Three objectives were used for the Boost in an analogous manner. For the rectum four objective values described the desired course of the DVH. No constraints were set for the optimization, rather objectives were appropriately weighted: the weights for the objectives were chosen in a wide range from 0.1 (i. e. for the shells of healthy tissue surrounding the PTV) to 100 (i. e. for PTV dose minima). The BEV view of the SOIs for the gantry at around 270° is shown in Figure 4. The set of pre-DMPO segments for Boost and PTV is shown in Figure 5, top. The same segments after DMPO fine-tuning are shown in Figure 5, bottom.

Patients were advised to have an empty bowel and a full bladder for the planning CT and during the treatment. For cone-beam CT (CBCT) of the day, the standard clinical protocol based on the soft tissue/bony anatomy alignment is applied. The patient is relocated to achieve maximal overlap between the PTV/Boost and the prostate as seen in the CBCT image. The rectum and the bladder should remain reasonably spared. In approximately 2% of cases the matching between the planning and daily structures is unsuccessful, and the patients are sent to undergo a repeated planning CT. The SOIs are always contoured by the same attending physician according to well predefined rules. All clinical CTs are reconstructed with a voxel size of 1 mm in-plane and 2.5 mm slice thickness.

Five intermediate/high-risk prostate carcinoma patients for which it was not possible to match the planning SOIs to the CBCT image on the day of treatment were selected for the present study. In the following, CT1 refers to the initial planning CT, CT2 refers to the repeated planning CT.

**Figure 6:**
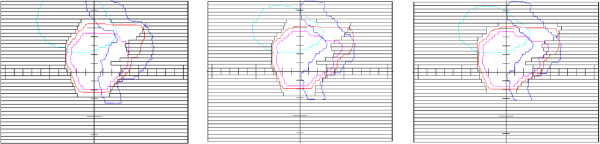
Adaptation example: left – S0 segment for the PTV, fine-tuned for CT1; middle – the same segment in CT2 after isocenter relocation; right – corresponding adapted segment.

In this work we adapted both PTV and Boost segments to be able to adequately compare the dose distributions of the adapted plans and the native plans using our standard clinical protocols. A specific example of segment adaptation is shown in Figure 6. Figure 6, left, shows the S0 segment for the PTV from Figure 5, a, fine-tuned for CT1. Figure 6, middle, shows the same segment in CT2 after isocenter relocation. The CT1 S0 segment extends too much vertically and underexposes the PTV too much on the right. The vertical extension is taken into account at 2-Step generation, the geometric features from DMPO are re-introduced by DMPO imitation; the adapted segment is shown in Figure 6, right.

**Figure 7:**
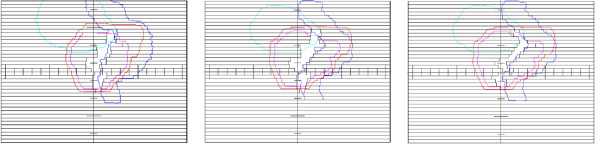
Adaptation example: left – S2 segment for the Boost, fine-tuned for CT1; middle – the same segment in CT2 after isocenter relocation; right – corresponding adapted segment.

Another specific example is shown in Figure 7, now for the S2 type segment from Figure 5, f, oriented at the Boost. Again, the geometric features of the segment edge adjacent to the rectum are re-introduced at DMPO imitation phase. The leaf openings are adapted to the changed 3D geometry in the corresponding CT layer: the rectum radius decreases in almost all CT layers, and leaf openings correspondingly decrease according to (1) and [14].

## Results

The dose distributions for all native, relocated, and adapted plans were calculated in Pinnacle^3^™. The evaluation of the obtained dose distributions was then performed. We measured target coverage using D_99%_ in the CTV and the quality score *S*_*D*_. The quality score *S*_*D*_ was introduced by the QUASIMODO group to compare different plans against the same dose objectives [15]. For our purposes *S*_*D*_ sums up violations of dose requirements for Boost and PTV - Boost:

(2) $$S_{D}=\sum_{j}\left\{\begin{array}{c} \left|A_{j}-R_{j\;\mathrm{lo}}\right|,\;\mathrm{if}\;A_{j}<R_{j\;\mathrm{lo}}\;\\ \left|A_{j}-R_{j\;\mathrm{up}}\right|,\;\mathrm{if}\;A_{j}>R_{j\;\mathrm{up}}\\ 0,\;\mathrm{if}\;R_{j\;\mathrm{lo}}\leq A_{j}\leq R_{j\;\mathrm{up}}\; \end{array}\right)$$

where *A*_*j*_ are achieved dose values, *R*_*j*__lo/up_ is the lower (upper) limit of the required dose range.

The following parameters characterized the course of the Boost DVH: D_mean_, D_99%_, D_95%_, D_1%_; of the PTV - Boost DVH: D_99%_, D_95%._ The dose ranges required for native plans can be found in Table 1. The plan which fulfils all requirements has *S*_*D*_ = 0. To evaluate dose load to the rectum we used absolute rectum volumes defined by the respective 50%, 80%, and 95% isodoses.

**Table 1:** **Evaluation for the case of the least volumetric difference between target SOIs in CT1 and CT2 (DVHs in Figure **8**, left)**

		**Required**	**Relocation**		**Native**		**Adapted**	
MUs			493		502		493	
CTV	D_99%_ [Gy]		62.7		64.3		68.7	
			achieved	*S*_*D*_	achieved	*S*_*D*_	achieved	*S*_*D*_
Boost								
	D_mean_ [Gy]	76.2 ± 1%:	75.5	0	-	-	76.1	0
		[75.4, 77.0]						
	D_99%_ [Gy]	>70	64.3	5.7	71.3	0	68.2	1.8
	D_95%_ [Gy]	74 ± 2%:	69.1	3.4	73	0	71.3	1.2
		[72.5, 75.5]						
	D_1%_ [Gy]	<80	79.6	0	79.1	0	81.9	1.9
PTV - Boost								
	D_99%_ [Gy]	>56	48.3	7.6	58.9	0	53.6	2.4
	D_95%_ [Gy]	60.1 ± 2%:	56.5	2.4	61.4	0.1	58.8	0.1
		[58.9, 61.3]						
Net *S*_*D*_				19.1		0.1		7.4
Rectum								
	V_95%_ [cm^3^]		2.8		1		0.1	
	V_80%_ [cm^3^]		9.1		9.1		5	
	V_50%_ [cm^3^]		26.1		27.8		27.2	

**Figure 8:**
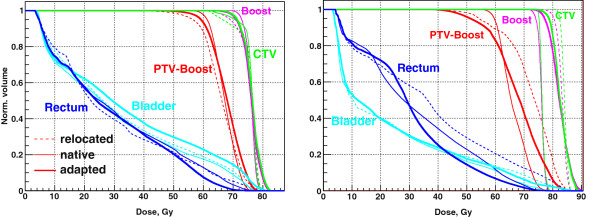
Adaptation for the least (left), and the largest (right) volumetric difference between target SOIs in CT1 and CT2: DVHs for relocated (thin dashed), native (thin solid), and adapted (thick solid) plans.

The change of the prostate volume between treatment fractions observed by [12] was 5-10%; only once did the authors observe a change of 20%. In Figure 8, left, and Table 1 we show the result of the adaptation for the case of the least volumetric difference between target SOIs in CT1 and CT2 which we observed, of the order of 5%; the corresponding difference between the MUs for the native plans is around 2%. Figure 8, left, presents the DVHs for relocated, native, and adapted plans. The visual comparison of the DVHs shows the improvement of the adapted plan against the relocated plan, both in terms of target coverage and rectum sparing. The results of DVH evaluation are shown in Table 1. The required dose values, achieved dose values, and the corresponding *S*_*D*_ calculated according to (2) are shown for relocated, native, and adapted plans. The improvement of the target coverage is reflected by the drop of *S*_*D*_ for the adapted plan against the relocated plan from 19.1 to 7.4 Gy. The D_99%_ for the CTV DVH increases correspondingly. The rectum sparing for the adapted plan considerably improves against the relocated plan, as V_95%_ and V_80%_ values indicate.

**Figure 9:**
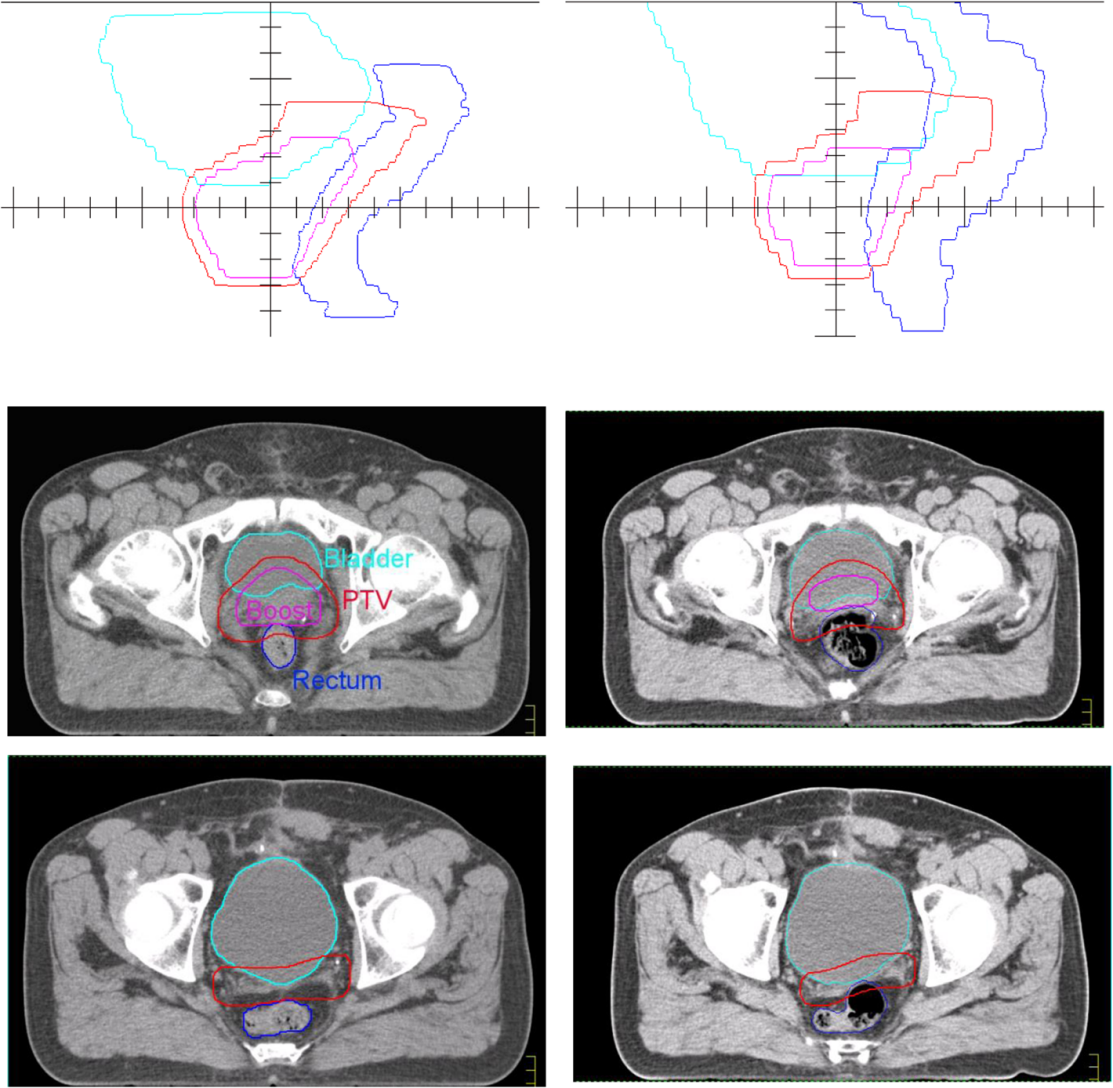
The prostate case of the largest volumetric difference between target SOIs in CT1 (left) and CT2 (right): top – BEVs; middle, bottom – CT layers showing an effect of increased bladder and rectum filling on Boost and PTV contours.

The adaptation result for the case with the largest volumetric difference between target SOIs in CT1 and CT2 is shown in Figure 8, right. Due to increased bladder and rectum filling in CT2 (Figure 9, top), the prostate is pushed down and the seminal vesicles are compressed between the bladder and the rectum. Consequently, the upper part of the PTV which encloses the base of the seminal vesicles extends further up in CT2 compared to CT1, and the Boost shrinks from 89 to 70 cm^3^, by 25%, as compared to much more moderate difference of Boost volumes for other patients, of the order of several percent. Additionally, the rectum folds in some CT2 layers in (Figure 9, bottom). As a result, the plans optimized for native geometries require 697 and 593 MUs, with the difference of the order of 15%, as compared to the difference of the order of 2% for other four patients. As DVHs in Figure 8, right, and corresponding evaluation in Table 2 show, the result of the adaptation to such a different geometry is marginal. The coverage gets even worse: *S*_*D*_ for the adapted plan increases against the relocated plan from 31.5 to 36.8 Gy; however, rectum sparing improves considerably.

**Table 2:** **Evaluation for the case of the largest volumetric difference between SOIs in CT1 and CT2 (DVHs in Figure **8**, right)**

		**Required**	**Relocation**		**Native**		**Adapted**	
MUs			697		593		697	
CTV	D_99%_ [Gy]		81.7		75		76.4	
			achieved	*S*_*D*_	achieved	*S*_*D*_	achieved	*S*_*D*_
Boost								
	D_mean_ [Gy]	76.2 ± 1%:	83.3	6.3	-	-	82.5	5.5
		[75.4, 77.0]						
	D_99%_ [Gy]	>70	78.7	0	73	0	75.3	0
	D_95%_ [Gy]	74 ± 2%:	80.5	5.0	74.2	0	77.2	1.7
		[72.5, 75.5]						
	D_1%_ [Gy]	<80	86.1	6.1	78.5	0	90.1	10.1
PTV - Boost								
	D_99%_ [Gy]	>56	46.4	9.6	58	0	44	12
	D_95%_ [Gy]	60.1 ± 2%:	54.4	4.5	59.7	0	51.4	7.5
		[58.9, 61.3]						
Net *S*_*D*_				31.5		0		36.8
Rectum								
	V_95%_ [cm^3^]		14		1.7		1	
	V_80%_ [cm^3^]		24.5		13.2		7.5	
	V_50%_ [cm^3^]		58.3		46.6		35.3	

Two CTs per case provide two adaptation directions, from CT1 to CT2 (“forward”) and from CT2 to CT1 (“backward”). We considered “forward” and “backward” adaptations as independent, so five patients provide ten adaptation cases. Table 3 shows the target coverage characterized by the *S*_*D*_ value, and the dose load to the rectum characterized by the V_95%_ value for all ten cases. According to Marzi et al. [21] V_95%_ ≡ V_72,4Gy_ is highly correlated with the NTCP of the late rectal bleeding. We compared pairwise *S*_*D*_ and V_95%_ of adapted plans against relocated, native plans against adapted, and native plans against relocated, using 1-tail Wilcoxon signed rank test. The resulting Wilcoxon probabilities are shown in Table 4. As one can see, adapted plans provide a significant improvement (Wilcoxon *p* < 0.05) of both the target coverage and the rectum sparing against relocated plans.

**Table 3:** **The *S***_***D ***_**and rectum V**_**95% **_**values for ten adaptation cases**

**Case**	***S***_***D***_**,Gy**			**V**_**95%**_**, cm**^**3**^		
	**Relocated**	**Native**	**Adapted**	**Relocated**	**Native**	**Adapted**
1	74.1	17.8	41.1	2.8	0.2	0.1
2	63.1	5.4	5.1	14	0.6	2.9
3	19.2	5.7	13.4	4.4	2.4	4.5
4	19.1	0.1	7.4	2.8	1	0.1
5	45.2	0	5.3	0	1.4	3
6	31.5	0	36.8	14	1.7	1
7	67.8	1	9.6	2	2.7	0.1
8	83.3	0	17.1	7.3	1.8	3.3
9	23.5	0,5	11.5	17.7	6	4.3
10	69.8	0	24.5	0.2	2.4	0.4

**Table 4:** **The results of the 1-tail Wilcoxon signed rank test for *S***_***D ***_**and rectum V**_**95% **_**from Table**3**, different plan groups are compared (see text for explanation)**

	**Wilcoxon** ***p***	
	***S***_***D***_	**V**_**95%**_
Adapted against relocated	0.0037*	0.0314*
Native against adapted	0.0037*	0.4483
Native against relocated	0.0027*	0.0250*

(*) marks significant difference (*p* < 0.05).

## Discussion

**Figure 10:**
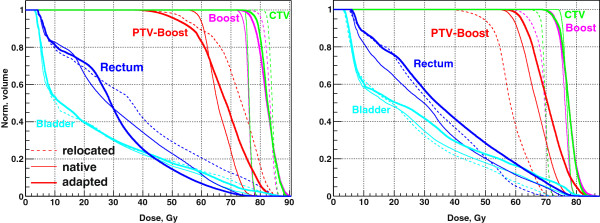
**Adaptation for the case of the largest volumetric difference between target SOIs in CT1 and CT2: forward direction (left) and backward direction (right).** Either target coverage or rectum sparing is compromised after adaptation.

The prostate case with large volumetric difference between CTs, and, correspondingly, with large MU difference between native plans, of the order of 15%, deserves special attention. Figure 10, left, reproduces the result from Figure 8, right. Figure 10, right, shows for comparison the result of the adaptation in the opposite direction, from CT2, Figure 9, right, to CT1, Figure 9, left. The adaptation in the opposite direction produces a plan with much better target coverage characterized by the drop from *S*_*D*_ = 45.2 Gy to *S*_*D*_ = 5.3 Gy, but marginally acceptable rectum sparing characterized by V95% = 3 cm^3^. So either target coverage or rectum sparing is compromised after forward or backward adaptation. This case gives an indication of “large” interfractional geometry difference: volumetric differences on the order of 25% (MU difference on the order of 15%) are likely to bring the 2-Step adaptation method to its limits. Such large interfractional differences are, however, expected to be rare in clinical practice [12]. For such cases the extension to MU-modifying adaptation seems to be necessary [16].

## Conclusions

The present work reminds the reader the principles of the 2-Step IMRT adaptation, for the first time introduced in [13], and manually applied to the prostate cases in [14]. The 2-Step IMRT adaptation to the changed geometry of the QUASIMODO phantom has been considered for illustrative purposes. The automated 2-Step IMRT adaptation for ten prostate carcinoma adaptation cases has been performed. The adapted plans show statistically significant improvement (Wilcoxon 1-tail *p* < 0.05) of the target coverage and of the rectum sparing against the relocated plans.

This paper provides present status of the on-going work towards developing an adaptive radiation therapy (ART) system at UKW. Next, the potential margin reduction has to be investigated. Monitoring of delivered fraction dose has to be implemented, e.g. using post-fraction summation of the dose distributions based on the non-rigid registration; this would allow falling back to complete re-planning in the case of threatening rectum overdosage or target underdosage. The standalone DICOM-aware adaptation system is planned to be developed, supplemented by the efficient quality assurance system for ART plans.

## Appendix

### Appendix A. 2-Step segmentation

The principle behind 2-Step segmentation stems from the fundamental work of Brahme [18] and is described in detail elsewhere [22]. In short, it uses geometric analysis of structures of interest to generate segments which deliver three fluence steps: conformal to the target (Step 0, “S0”), sparing the OAR (Step 1, “S1”), and narrow segments compensating for the underdosage in the target due to OAR sparing (Step 2, “S2”). The width of the narrow S2 segments is calculated for every MLC leaf pair based on the target and rectum geometry in the corresponding CT layer to have best target coverage as explained in [13].

**Figure 11:**
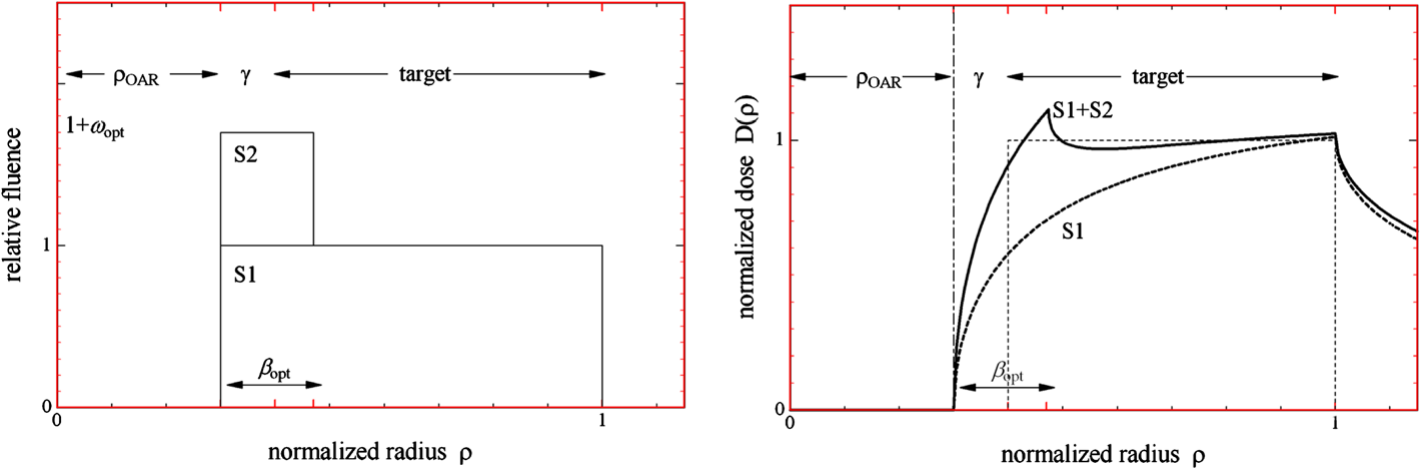
**S1 and S2 fluence steps (left) which produce approximately homogeneous dose distribution in the target (right, dotted line represents the ideal dose distribution).** Conformal S0 step defines the overall dose level and is omitted for clarity.

Two blocked rotations which were subsequently summed were considered, Figure 11. The resulting dose distribution is the sum of the solutions for a single blocked rotation discussed in [23]:

$$D\left(\rho_{\text{OAR}}\text{,}\;\beta^{\text{gen}}\text{,}\;\rho \right)=D\left(\rho_{\text{OAR}}\text{,}\;1\text{,}\;\rho \right)+\omega \cdot D\left(\rho_{\text{OAR}}\text{,}\;\beta^{\text{gen}}\text{,}\;\rho \right)$$

where *ρ* = *r*/*R*_PTV_ is the normalized radial coordinate, *ρ*_OAR_ = *R*_OAR_/*R*_PTV_ is the normalized OAR radius, *ω* is the weight of the second order blocking segment relative to the first order blocking segment (normalized weight), *β* = *w*/*R*_PTV_ is the normalized width of the second order blocking segment.

To find an optimal normalized width of the second order blocking segment which best homogenizes the dose distribution in the target, *β*_opt_, one has to minimize the objective function

(A.1) $$f^{2}=\sigma_{D}^{2}+{\left(D-D_{0}\right)}^{2},$$

where *D*_0_ is the prescribed dose in the target, *σ*_*D*_ is the root mean square of the dose distribution in the target. It has been shown that the pair of optimal normalized weight and width [*ω*_opt_, *β*_opt_] for the second order blocking segment always exists, Figure 11, and the finding was that the product *ω*_opt_ ⋅ *β*_opt_ ≡ β* depends only weakly on the normalized target-OAR gap *γ*: $\beta^{*}=\beta_{\gamma }^{*}\left(\rho_{\mathrm{OAR}}\right)$. The set of curves $\beta_{\gamma }^{*}\left(\rho_{\mathrm{OAR}}\right)$ was found analytically. It can be used to find *β*_opt_ for any geometry assuming *ω* = 1.

The 2-Step generation is a forward planning technique and does not take detailed spatial relationship of the organ constellation into account. Therefore it is followed by the inverse planning Direct Machine Parameter Optimization (DMPO) stage to fine-tune segment shapes and weights. The DMPO essentially performs second-order correction on top of the 2-Step segment set. The equivalence of DMPO-fine-tuned 2-Step IMRT plans and conventional IMRT plans, for example as realized by the DMPO™ module of Pinnacle™, has been shown in earlier publications [24].

## Competing interests

The authors declare that they have no competing interests.

## Authors’ contributions

KB is the author of the idea of 2-Step IMRT segmentation and adaptation, and is the primary author of the previous papers on the topic. MG and KH developed the software for 2-Step segmentation and adaptation. KH drafted the manuscript; MG and KB critically accompanied and revised the manuscript. BP and MF provided the patient sample, performed the contouring, and critically accompanied the study. All authors read and approved the final manuscript.
